# Hybrid Compounds Strategy in the Synthesis of Oleanolic Acid Skeleton-NSAID Derivatives

**DOI:** 10.3390/molecules21040420

**Published:** 2016-04-12

**Authors:** Anna Pawełczyk, Dorota Olender, Katarzyna Sowa-Kasprzak, Lucjusz Zaprutko

**Affiliations:** Department of Organic Chemistry, Pharmaceutical Faculty, Poznan University of Medical Sciences, Grunwaldzka 6, 60-780 Poznań, Poland; dolender@ump.edu.pl (D.O.); kasik@ump.edu.pl (K.S.-K.); zaprutko@ump.edu.pl (L.Z.)

**Keywords:** oleanolic acid, oleanolic oxime, NSAIDs, ibuprofen, aspirin, naproxen, ketoprofen, hybrid compounds

## Abstract

The current study focuses on the synthesis of several hybrid individuals combining a natural oleanolic acid skeleton and synthetic nonsteroidal anti-inflammatory drug moieties (NSAIDs). It studied structural modifications of the oleanolic acid structure by use of the direct reactivity of hydroxyl or hydroxyimino groups at position C-3 of the triterpenoid skeleton with the carboxylic function of anti-inflammatory drugs leading to new perspective compounds with high potential pharmacological activities. Novel ester- and iminoester-type derivatives of oleanolic unit with the different NSAIDs, such as ibuprofen, aspirin, naproxen, and ketoprofen, were obtained and characterized. Moreover, preliminary research of compounds obtaining structure stability under acidic conditions was examined and the PASS method of prediction of activity spectra for substances was used to estimate the potential biological activity of these compounds.

## 1. Introduction

**Figure 1:**
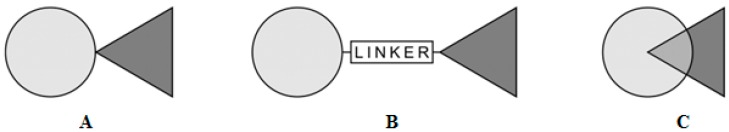
General integration modes of active dual structures. (**A**) Direct no-linker mode (fused hybrids); (**B**) Intermediate linker mode; (**C**) Overlap mode (merged hybrids).

Many scientific reports on the future prospects for the design of potentially useful drugs have been published. Among the many synthetic trends (dimers, heterodimers, heteromers, biooligomers, dendrimers, dual-, bivalent- or multifunction agents, identical or non-identical twin drugs, mixed or combo drugs, supramolecular particles, and various nanoindividuals) a clear leader is a hybrid strategy [1]. A hybrid compound is a synthetic product combining two or more separate active structures (or structure fragments) joined by at least one chemical bond. The unlimited possibilities of combining natural or unnatural active compounds can be exploited to generate individuals with unprecedented bioactivity and an additional potential synergic effect. Chemical association of two structures into one new dual molecule depends on their functionality and cross-reactivity, and can be implemented by way of various methods [2,3]; Generally, the most popular molecules integration modes are: direct no-linker mode (fused hybrids) A; intermediate linker mode, B; and overlap mode (merged hybrids), C (Figure 1). The use of hybrids for disease treatment could offer several advantages, such as increased solubility, and several mechanisms of action in one molecule. This approach can be used to optimize certain biological properties, like affinity and selectivity, but also to produce novel biological activities distinct from those of the components.

Due to the great potential of natural products exhibiting pronounced biological activities, they have been one of the major sources of components in hybrid molecules and, in practice, at least one hybrid fragment should be natural. Terpenes are a widespread group of natural compounds with considerable practical significance, which are found in most plants, as a result of their wide spectrum of biological activities.

Inspired by the latest literature reports [4,5,6] as a key modulator of the activity of other biologically-active compounds (NSAIDs), we decided to use the broadly advantageous structure of naturally-occurring 3β-hydroxyolean-12-en-28-oic acid, commonly known as oleanolic acid (**1**). From a chemical point of view, it belongs to pentacyclic triterpene from the β-amyrine group with five six-membered cycles in the molecule, which offers great powers both as a bioactive molecule and as a carrier for other external activities. In nature, this compound exists either as a free acid or as a component of triterpenoid saponins, in which it can be linked with one or more sugar chains. This natural triterpene, widely distributed in plants, has shown several relevant biological activities including the modulation of the immune inflammatory response [7]. Oleanolic acid and its derivatives possess several promising pharmacological activities, such as hepatoprotective and anti-inflammatory, antiviral, antibacterial, fungicidal, antioxidant, or anticancer [8]. Therefore, terpenes containing the hydroxyl or hydroxyimino reactive groups can be used to prepare derivatives with the commercial anti-inflammatory drugs with a carboxylic function, leading to new dual compounds. Recently, its semi-synthetic acyloxyimino derivatives with analgesic and anti-inflammatory effects were also reported [9,10]. In the last decade NSAID drugs have been extensively investigated for anticancer and chemopreventive activity against various types of cancers [11,12]. They appear to act via depression of prostaglandin synthesis through inhibiting cyclooxygenase-2, which results in suppression of proliferation, possibly throught enhancement of apoptosis [13]. However, NSAID drugs are known for their side effects on the gastrointestinal system, particulary stomach ulceration, bleeding, perforation, and action exerted by contact of drug with gastric mucosa [14]. Inflammation is not direct, but very often the initial, trigger of several different diseases, it is supposed that fusing the anti-inflammatory drugs and the oleanane active structure, resulting in combined compounds, showed an enhanced biological effect with synergistic action and minimized toxicity or adverse reactions.

## 2. Results and Discussion

**Scheme 1:**
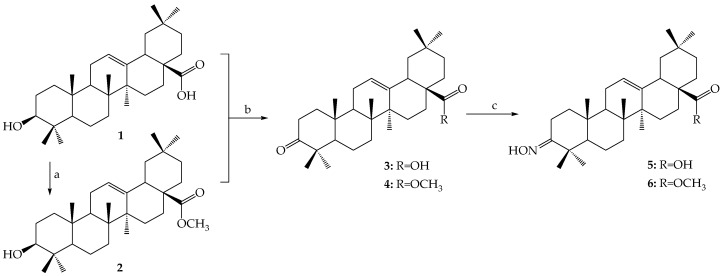
Oleanolic acid transformations. *Reagents and condition*: (a) (CH_3_)_2_SO_4_, NaOH, EtOH, reflux; (b) Jones reagent, acetone, RT; and(c) NH_2_OH × HCl, CH_3_COONa, EtOH, reflux.

Our research examined the reactivity of oleanolic acid (**1**), oleanolic acid methyl ester (**2**), and their corresponding oxime derivatives (**5**, **6**) in the creation of new dual compounds. In order to obtain the starting reactants, the carboxylic group (C-28) of oleanolic acid (**1**) was converted into the methyl ester group (**2**) by use of dimethyl sulfate in an ethanolic solution of sodium hydroxide as a methylation agent [15]. The secondary hydroxyl group at the C-3 position of the oleanolic acid skeleton was reacted in the oxidation process with chromic anhydride in sulfuric acid (Jones reagent) to give 3-oxooleanolic derivatives (**3**, **4**) [16]. The carbonyl derivatives formed at the C-3 position were reacted with hydroxylamine hydrochloride in the presence of sodium acetate and converted to corresponding oximes (**5**, **6**) [17] (Scheme 1).

**Figure 2:**
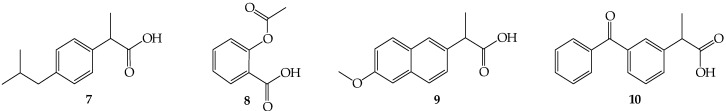
NSAID molecules selected.

The triterpene compounds mentioned above (**1**, **2**, **5**, **6**) are useful starting reagents in the synthesis of oleanolic acid skeleton-NSAID hybrid derivatives. Racemic compounds, such as ibuprofen (**7**), aspirin (**8**), naproxen (**9**), and ketoprofen (**10**) were applied as non-steroidal anti-inflammatory particles (Figure 2).

**Scheme 2:**
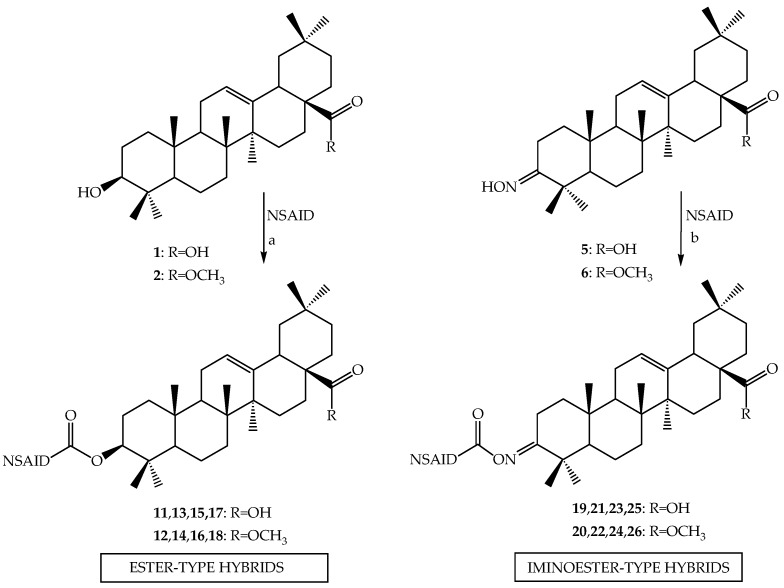
The main chemical directions of oleanolic acid-NSAID compounds synthesis. *Reagents and condition*: (a) ester-type hybrids: DCC, DMAP, anhyd. CHCl_3_, RT; and (b) iminoester-type hybrid: DCC, anhyd. CHCl_3_, RT.

The main transformation planned to afford hybrid derivatives is a reaction of the secondary hydroxyl group, or hydroxyimino group resulting from it, which is present in the terpene molecule at the C-3 position with a free carboxylic moiety of the anti-inflammatory agents selected. These move in two chemical directions (Scheme 2). Compounds **1** and **2** reacted by the esterification method lead to the formation of ester-type hybrid derivatives (**11**–**18**), while the corresponding terpene oximes (**5**,**6**) give an ester of oximes called as iminoester-type hybrid derivatives (**19**–**26**).

Oleanolic acid (**1**) and its methyl ester **2** were reacted respectively with appropriate NSAID compounds (**7**–**10**) in the presence of dicyclohexylcarbodiimide (DCC) and 4-(dimethylamino) pyridine (DMAP) reagents. DCC is carboxylic acid activating and coupling agent, while strongly-nucleophilic DMAP acts as an acyl transfer reagent in the Steglich esterification reaction [5,6] which is a very useful process in the conversion of sterically-demanding substrates. The water resulting from a DCC mediated reaction reacts immediately with the carboimide applied and forms dicyclohexylurea (DHU), which is insoluble in a reaction medium and precipitates as a white solid. It is, therefore, essential to carry out the reaction in anhydrous conditions. High DMAP addition is also crucial for the efficient formation of esters because in this case catalytic amounts of DMAP does not yield the product expected. The molar ratio of terpene alcohol, NSAID, DCC, and DMAP reagents used in the reaction process is in the sequence 1:4:5:3. In the case of the reaction of terpene oximes (**5**, **6**) with carboxylic anti-inflammatory agents (**7**–**10**) towards oxime esters a similar procedure was applied, but the presence of DMAP is not crucial. The molar ratio of reagents used was 1:4:5 (terpene oxime, NSAID, and DCC). All reactions, both towards ester and iminoester-type compounds, were carried out in an anhydrous chloroform solution. The reagents were mixed together at 0 °C, and then stirred at room temperature for up to two hours to achieve full conversion of the terpene substrate. Due to the fact that, during the reaction, a urea derivative is precipitated as byproduct, the addition of hexane and cooling the reaction mixture before filtration give the best final results, because the DHU is removed completely [5] (Scheme 2).

**Figure 3:**
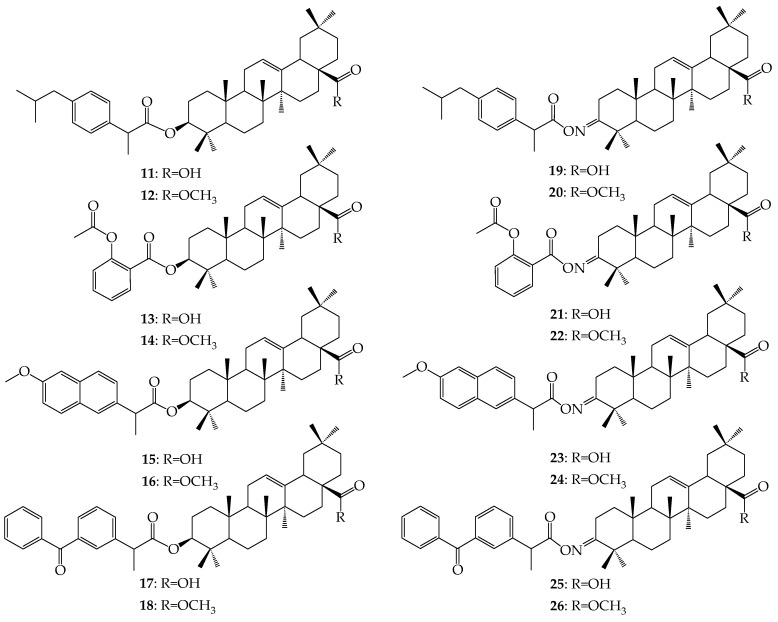
Formulas of the final hybrid derivatives obtained.

As a result of synthesis, sixteen structures hybridized with the formulas shown in Figure 3 were produced. Further isolation and purification by column chromatography resulted in the desired pure esters **11**–**18** and iminoesters **19**–**26**, with yields ranging from 62% to 89%. The structure of these hybrid compounds **11**–**26** was confirmed by ^1^H- and ^13^C-NMR, DEPT, and MS data.

### 2.1. Preliminary Stability Studies

New potential therapeutic substances are susceptible to various transformations in human body conditions. Compounds obtained as complex ester or iminoester individuals formed from triterpene and anti-inflammatory units under suitable conditions may disintegrate into the compounds from which they originated. Preliminary stability studies on the above compounds, in conditions outside the human organism, were carried out using acidic hydrolysis conditions with hydrochloric acid in an ethanolic solution (pH = 5). Compounds selected from each group, four esters (**11**, **13**, **15**, **17**) and four iminoesters (**19**, **21**, **23**, **25**) were treated with an acidic ethanol solution and stirred. The test was conducted at room temperature, and then in the case of stable compounds, at an elevated temperature (50 °C). Stability control of the test compounds was performed with the TLC method using structural reference substances after 1, 5, and 24 h. The tests clearly show that the resulting compounds are stable under the conditions employed and do not lead to a substrate or do not show changes in its structure, either at room temperature or at an elevated temperature. The results obtained are the initial data on the stability of the esters and iminoesters studied which in the body, under the action of enzymes, does not have to be stable. It is known that ester links are generally cleaved in the body by esterase leading to the starting active components.

### 2.2. Prediction of Activity Spectra by the PASS Method

In this study, to estimate the biological activity of the substances obtained, the PASS method (Prediction of Activity Spectra for Substance) was used. PASS Online [18] predicts over 4000 kinds of biological activity, including pharmacological effects, mechanisms of action, toxic and adverse effects, interaction with metabolic enzymes and transporters, or the influence on gene expression.

Biological activity, the P_a_ factor (probability “to be active”) and P_i_ factor (probability “to be inactive”) spectrum for NSAID-triterpene derivatives using the structure-activity relationships were calculated and predicted. All substances tested **11**–**26** showed a broad spectrum of potential activity. Most of these compounds exhibit a very high probability of biological activity, with more than 70% occurring. Table 1 and Table 2 summarize the results predicted biological activities and potential mechanisms of action derivatives obtained.

If the value of P_a_ factor is greater than 70%, there is a high probability that the substance will be active in the conditions of *in vitro* experiment, whereas if the P_a_ factor is within the 50%–70% range, there is a slightly lower probability confirming the activity of the substance in the biological studies. Therefore, in Table 1 and Table 2 only the results for the highest value of the P_a_ factor above 70% were presented. Ester-type connections, compared to the corresponding iminoester-type compounds, are potentially more active individuals with a much higher P_a_ factor above 70%. Related iminoesters also demonstrate potential activity, although many of them are in the P_a_ factor range of 50%–70%.

The resulting derivatives, particularly ester-type compounds, have a high potential to become effective pharmaceutical individuals: e.g., as new anti-inflammatory agents that may show a remarkable increase in activity and decrease toxicity, compared with a single synthetic anti-inflammatory drug or terpene. Analyzing the data obtained using the PASS method, it is believed that the formation of both ester and iminoester moieties is certainly a good and useful strategy in the design of potential new therapeutic compounds that will minimize disease-associated morbidity and mortality.

## 3. Materials and Methods

### 3.1. Equipment and General Procedures

The melting points of all compounds used in this study were determined on a Boetius apparatus and were uncorrected. The ^1^H- and ^13^C-NMR spectra were recorded using a Varian Gemini 300 VT spectrometer, 300 and 75 MHz respectively (Agilent Technologies, Santa Clara, CA, USA). Chemical shifts (δ) were expressed in parts per million (ppm), relative to tetramethylsilane (TMS) as an internal standard, using CDCl_3_ as solvent. Coupling constants (*J*) are expressed in Hertz (Hz). Splitting patterns are designated as follows: s, singlet; d, doublet; dd, double doublet; t, triplet; and m, multiplet.

MS spectra were recorded on a 402 AMD INTECTRA apparatus (AMD Intectra GmbH, Harpstedt, Germany) by the electron impact technique (EI), operating at 75 eV. The infrared (IR) spectra were recorded in KBr tablets using a Specord 75–IR spectrophotometer (Carl Zeiss, Jena, Germany) and were expressed in the cm^−1^ scale. The progress of reactions and purity of products were checked using the TLC method on silica gel plates (60 F_254_ from Merck, Darmstadt, Germany) in hexane/ethyl acetate (4:1, *v*/*v*) as a developing system. The TLC spots on the plates were observed in UV light (λ = 254 nm) and were visualized by spraying the chromatograms with 10% H_2_SO_4_ in ethanol and heating the plates at 110 °C for approximately 3 min. Silica gel 60 (63–200 μm particle size, Merck) was used for column chromatography.

Oleanolic acid (Bio-Tech, Beijing, China, plant material: *Olea europea*), DCC, DMAP, racemic NSAID compounds (ibuprofen, aspirin naproxen, ketoprofen), and solvents (chloroform, ethyl acetate, hexane) were commercial products (Sigma-Aldrich, St. Louis, MO, USA). The compounds used for the synthesis were of a purity higher than 98%. Chloroform was additionally dried over anhydrous CaCl_2_ and distilled. Other solvents were used without further purification.

### 3.2. Preparation of Oleanolic Acid- and Their Me-Ester-NSAID Hybrid Derivatives

#### 3.2.1. Starting Oleanolic Acid Derivatives **2**–**6**

First, oleanolic acid (**1**) was methylated with dimethyl sulfate in an ethanolic solution of NaOH and methyl oleanolate (**2**) was obtained with a high yield [19]. Next, oleanolic acid and its methyl ester were oxidized with Jones reagent in acetone at room temperature [16]. The 3-oxoderivatives obtained (**3**, **4**) were subjected to a reaction with hydroxylamine hydrochloride in ethanol, in accordance with the procedure known for many different oxotriterpenoids. Heating oleanolic ketones (**3**, **4**) with hydroxylamine hydrochloride produces the corresponding oleanolate oximes (**5**, **6**) [17]. The spectral data of all the starting compounds (**2**–**6**) were confirmed the data from the literature [16,17,19,20].

#### 3.2.2. Synthesis of Ester-Type Hybrid Compounds **11**–**18**

Oleanolic acid (**1**) or oleanolic acid methyl ester (**2**) (1 mmol), DCC (1.03 g, 5 mmol), DMAP (0.37 g, 3 mmol) and anti-inflammatory drugs: ibuprofen, aspirin, naproxen, or ketoprofen (4 mmol) were mixed at 0 °C and then stirred at room temperature in dry CHCl_3_ (12 mL) for 2 h. Hexane (8 mL) was added to the reaction mixture and cooled. Next, the resulting precipitate of dicyclohexylurea was filtered. The organic phase was washed with 5% aqueous hydrochloric acid, 5% aqueous sodium bicarbonate and water and dried over anhydrous sodium sulfate. After removal of the drying agent and solvent evaporation, the crude new esters (**11**–**18**) were purified with column chromatography on silica gel using a hexane–ethyl acetate (4:1, *v*/*v*) mixture as an eluent.

*Oleanoyl ibuprofenate* (**11**) [4]: after purification by silica gel column chromatography yield: 0.56 g (87%) of **11** (R_f_ = 0.48, hexane**/**EtOAc 4:1) as colorless solid, mp: 228–230 °C; IR ν_max_ (KBr, cm^−1^) = 3300–3420 (COOH), 1710 (C=O, ester ibu-olean), 1695 (COOH), 1170 (C–O, ester ibu-olean); ^1^H-NMR (CDCl_3_): Oleanoyl moiety: δ = 11.01 (1H, s, COOH), 5.26 (1H, t, *J* = 3.4, CH-12), 4.43 (1H, m, *C*H*-*3), 2.80 (1H, dd, *J* = 14.1, 3.4, CH-18), 1.11–0.54 (21H, s, 7 × CH_3_); Ibuprofen moiety: δ = 7.20 (2H, d, *J* = 8.4, CH-arom), 7.07 (2H, d, *J* = 8.0, CH-arom), 3.69 (1H, m, CH), 2.44 (2H, d, *J* = 4.0, CH_2_), 2.25 (1H, m, CH), 1.49 (3H, d, *J* = 7.0, CH_3_), 0.87 (3H, s, CH_3_), 0.86 (3H, s, CH_3_); ^13^C-NMR (CDCl_3_): Oleanoyl moiety: δ = 183.50 (C-28), 143.58 (C-13), 122.53 (C-12), 80.85 (C-3), 55.30 (C-5), 47.60 (C-9), 46.72 (C-17), 45.85 (C-19), 41.72 (C-14), 41.30 (C-18), 39.50 (C-8), 38.10 (C-1), 37.90 (C-4), 36.91 (C-10), 33.88 (C-21), 33.15 (C-29), 32.70 (C-7), 32.52 (C-22), 30.70 (C-20), 29.81 (C-15), 27.96 (C-23), 27.80 (C-2), 25.98 (C-27), 23.66 (C-30), 23.55 (C-16), 23.45 (C-11), 18.20 (C-6), 17.88 (C-26), 16.85 (C-24), 15.30 (C-25); Ibuprofen moiety: δ = 174.35 (C=O ester ibu-olean), 140.39, 137.74, 137.48, 129.25, 127.33, 127.10 (6 × C-arom), 44.93 (CH_2_), 41.59 (CHCOO), 27.55 (CH(CH_3_)), 22.30, 22.20, 19.60 (3 × CH_3_); DEPT: 10 × CH_3_, 11 × CH_2_, 11 × CH; MS (EI) *m/z =* 645.0 [M]^+^ (C_43_H_64_O_4_).

*Oleanoyl ibuprofenate methyl ester* (**12**) [4]: after purification by silica gel column chromatography yield: 0.53 g (80%) of **12** (R_f_ = 0.85 (hexane**/**EtOAc 4:1) as colorless solid, mp:139–141 °C; IR ν_max_ (KBr, cm^−1^) = 1720 (C=O, ester ibu-olean), 1700 (COOCH_3_), 1170 (C–O, ester ibu-olean); ^1^H-NMR (CDCl_3_): Oleanoyl moiety: δ = 5.27 (1H, t, *J* = 3.4, CH-12), 4.42 (1H, m, CH-3), 3.67 (3H, s, OCH_3_), 2.85 (1H, dd, *J* = 13.6, 3.3, CH-18), 1.11–0.54 (21H, s, 7 × CH_3_); Ibuprofen moiety: δ = 7.20 (2H, d, *J* = 8.4, CH-arom), 7.07 (2H, d, *J* = 8.0, CH-arom), 3.67 (1H, m, CH), 2.43 (2H, d, *J* = 4.0, CH_2_), 2.26 (1H, m, CH), 1.50 (3H, d, *J* = 7.0, CH_3_), 0.87 (3H, s, CH_3_), 0.86 (3H, s, CH_3_); ^13^C-NMR (CDCl_3_): Oleanoyl moiety: δ = 178.39 (C-28), 143.70 (C-13), 122.39 (C-12), 80.90 (C-3), 55.63 (C-5), 51.59 (COOCH_3_), 47.77 (C-9), 46.70 (C-17), 45.87 (C-19), 41.70 (C-14), 41.20 (C-18), 39.51 (C-8), 38.20 (C-1), 37.24 (C-4), 36.70 (C-10), 33.75 (C-21), 33.01 (C-29), 32.75 (C-7), 31.40 (C-22), 30.23 (C-20), 28.45 (C-15), 27.90 (C-23), 27.45 (C-2), 25.80 (C-27), 23.78 (C-30), 23.55 (C-16), 23.48 (C-11), 18.29 (C-6), 17.40 (C-26), 16.90 (C-24), 15.30 (C-25); Ibuprofen moiety: δ = 174.36 (C=O ester ibu-olean), 140.37, 137.73, 137.48, 129.10 127.25 (6 × C-arom), 127.08, 44.92 (CH_2_), 41.60 (CHCOO), 27.56 (CH(CH_3_)), 22.32, 22.30, 19.29 (3 × CH_3_); DEPT: 11 × CH_3_, 11 × CH_2_, 11 × CH; MS (EI) *m/z =* 658.6 [M]^+^ (C_44_H_66_O_4_).

*Oleanoyl aspirinate* (**13**): after purification by silica gel column chromatography yield: 0.38 g (62%) of **13** (R_f_ = 0.45, hexane**/**EtOAc 4:1) as colorless solid, mp: 182–184 °C; IR ν_max_ (KBr, cm^−1^) = 3350 (COOH), 1710 (C=O, ester asp-olean), 1700 (COOH), 1650 (C=O asp), 1175 (C–O, ester asp-olean); ^1^H-NMR (CDCl_3_): Oleanoyl moiety: δ = 11.01 (1H, COOH), 5.28 (1H, t, *J* = 3.4, CH-12), 4.49 (1H, m, CH-3), 2.86 (1H, dd, *J* = 14.1, 3.4, CH-18), 1.12–0.56 (21H, s, 7 × CH_3_); Aspirin moiety: δ = 7.50, 7.43, 7.22, 7.09 (4H, m, CH-arom), 2.33 (3H, s, CH_3_); ^13^C-NMR (CDCl_3_): Oleanoyl fragment: δ = 180.83 (C-28), 143.76 (C-13), 122.30 (C-12), 82.17 (C-3), 52.44 (C-5), 47.67 (C-9), 46.79 (C-17), 45.33 (C-19), 41.97 (C-14), 41.23 (C-18), 39.00 (C-8), 38.11 (C-1), 37.79 (C-4), 36.98 (C-10), 33.81 (C-21), 33.10 (C-29), 32.56 (C-22), 32.12 (C-7), 30.75 (C-20), 27.98 (C-15), 27.69 (C-23), 27.54 (C-2), 25.79 (C-27), 23.60 (C-30), 23.51 (C-16), 23.34 (C-11), 18.20 (C-6), 16.82 (C-26), 16.73 (C-24), 15.36 (C-25); Aspirin moiety: δ = 170.96 (C=O ester aspir-olean), 169.58 (COOCH_3_), 150.63, 137.33, 130.38, 125.80, 121.92, 116.13 (6 × Carom), 21.12 (CH_3_); DEPT: 8 × CH_3_, 10 × CH_2_, 9 × CH; MS (EI) *m/z =* 618. 9 [M]^+^ (C_39_H_54_O_6_).

*Oleanoyl aspirinate methyl ester* (**14**): after purification by silica gel column chromatography yield: 0.45 g (72%) of **14** (R_f_ = 0.65 (hexane**/**EtOAc 4:1) as colorless solid, mp: 223–226 °C; IR ν_max_ (KBr, cm^−1^) = 1725 (C=O, ester asp-olean), 1710 (COOCH_3_), 1660 (C=O asp), 1175 (C–O, ester asp-olean); ^1^H-NMR (CDCl_3_): Oleanoyl moiety: 5.28 (1H, t, *J* = 3.4, CH-12), 4.48 (1H, m, CH-3), 3.62 (3H, s, COOCH_3_), 2.85 (1H, dd, *J* = 13.6, 4.04, CH-18), 1.12–0.58 (21H, s, 7 × CH_3_); Aspirin moiety: δ = 7.52, 7.31, 7.21, 7.00 (4H, m, CH-arom), 2.06 (3H, s, CH_3_); ^13^C-NMR (CDCl_3_): Oleanoyl moiety: δ = 178.31 (C-28), 143.78 (C-13), 122.25 (C-12), 80.91 (C-3), 55.27 (C-5), 51.52 (COOCH_3_), 47.52 (C-9), 46.71 (C-17), 45.81 (C-19), 41.60 (C-14), 41.26 (C-18), 39.25 (C-8), 38.07 (C-1), 37.66 (C-4), 36.90 (C-10), 33.82 (C-21), 33.08 (C-29), 32.56 (C-7), 31.36 (C-22), 30.68 (C-20), 28.00 (C-15), 27.65 (C-23), 27.52 (C-2), 25.88 (C-27), 23.62 (C-30), 23.50 (C-16), 23.38 (C-11), 18.19 (C-6), 16.81 (C-26), 16.67 (C-24), 15.34 (C-25); Aspirin moiety: δ = 170.31 (C=O ester asp-olean), 170.04 (COOCH_3_), 150.04, 137.92, 131.38, 127.80, 121.99, 117.03 (6 × C-arom), 21.15 (CH_3_); DEPT: 9 × CH_3_, 10 × CH_2_, 9 × CH; MS (EI) *m/z =* 632.9 [M]^+^ (C_40_H_56_O_6_).

*Oleanoyl naproxenate* (**15**) [4]: after purification by silica gel column chromatography yield: 0.58 g (89%) of **15** (R_f_ = 0.44, hexane**/**EtOAc 4:1) as colorless solid, mp: 257–258 °C; IR ν_max_ (KBr, cm^−1^) = 3300–3420 (COOH), 1725 (C=O, ester napx-olean), 1690 (COOH), 1175 (C–O, ester napx-olean); ^1^H-NMR (CDCl_3_): Oleanoyl moiety: δ = 10.95 (1H, COOH), 5.25 (1H, t, *J* = 3.4, CH-12), 4.47 (1H, m, CH-3), 2.80 (1H, dd, *J* = 13.6, 4.0, CH-18), 1.11–0.57 (21H, s, 7 × CH_3_); Naproxen moiety: δ = 7.70 (1H, d, *J* = 8.8, CH-arom), 7.68 (1H, s, CH-arom), 7.64 (1H, d, *J* = 8.8, CH-arom), 7.42 (1H, d, *J* = 8.4, CH-arom), 7.40 (1H, d, *J* = 8.4, CH-arom), 7.14 (1H, d, *J* = 11, CH-arom), 3.91 (3H, s, OCH_3_), 3,84 (1H, m, CH), 1.57 (3H, d, *J* = 7.0, CH_3_); ^13^C-NMR (CDCl_3_): Oleanoyl moiety: δ = 183.24 (C-28), 143.53 (C-13), 122.51 (C-12), 81.06 (C-3), 55.26 (C-5), 47.46 (C-9), 46.49 (C-17), 45.79 (C-19), 41.56 (C-14), 40.95 (C-18), 39.21 (C-8), 37.98 (C-1), 37.84 (C-4), 36.89 (C-10), 33.76 (C-21), 33.02 (C-29), 32.48 (C-7), 32.39 (C-22), 30.63 (C-20), 29.72 (C-15), 27.68 (C-23), 27.61 (C-2), 25.85 (C-27), 23.53 (C-30), 23.49 (C-16), 23.35 (C-11), 18.17 (C-6), 17.99 (C-26), 16.52 (C-24), 15.27 (C-25); Naproxen moiety: δ = 174.25 (C=O ester napx-olean), 157.51, 136.02, 133.60, 129.23, 128.90, 127.46, 126.38, 125.99, 118.85, 105.56 (10 × C-arom), 55.3 (OCH_3_), 45.9 (CH), 17.04 (CH_3_); DEPT: 9 × CH_3_, 10 × CH_2_, 12 × CH; MS (EI) *m/z =* 668.5 [M]^+^ (C_44_H_60_O_5_).

*Oleanoyl naproxenate methyl ester* (**16**) [4]: after purification by silica gel column chromatography yield: 0.53 g (78%) of **16** (R_f_ = 0.60, hexane**/**EtOAc 4:1) as colorless solid, mp: 158–160 °C; IR ν_max_ (KBr, cm^−1^) = 1725 (C=O, ester napx-olean), 1700 (COOCH_3_), 1185 (C–O, ester napx-olean); ^1^H-NMR (CDCl_3_): Oleanoyl moiety: δ = 5.26 (1H, t, *J* = 3.6, CH-12), 4.46 (1H, m, CH-3), 3.61 (3H, s, COOCH_3_), 2.84 (1H, dd, *J* = 13.6, 4.0, CH-18), 1.10–0.56 (21H, s, 7 × CH_3_); Naproxen moiety: δ = 7.70 (1H, d, *J* = 8.8, CH-arom), 7.68 (1H, s, CH-arom), 7.67 (1H, d, *J* = 8.8, CH-arom), 7.42 (1H, d, *J* = 8.4, CH-arom), 7.40 (1H, d, *J* = 8.4, CH-arom), 7.14 (1H, d, *J* = 11, CH-arom), 3.91 (3H, s, OCH_3_), 3.83 (1H, m, CH), 1.58 (3H, d, *J* = 7.0, CH_3_); ^13^C-NMR (CDCl_3_): Oleanoyl moiety: δ = 178.30 (C-28), 143.77 (C-13), 122.23 (C-12), 81.04 (C-3), 55.27 (C-5), 51.51 (COOCH_3_), 47.46 (C-9), 46.69 (C-17), 45.96 (C-19), 41.59 (C-14), 41.25 (C-18), 39.22 (C-8), 38.00 (C-1), 37.84 (C-4), 36.86 (C-10), 33.82 (C-21), 33.08 (C-29), 32.51 (C-7), 32.35 (C-22), 30.67 (C-20), 29.69 (C-15), 27.67 (C-23), 27.63 (C-2), 25.87 (C-27), 23.62 (C-30), 23.50 (C-16), 23.39 (C-11), 18.15 (C-6), 16.78 (C-26), 16.54 (C-24), 15.27 (C-25); Naproxen moiety: δ = 174.25 (C=O ester napx-olean), 157.49, 136.02, 133.58, 129.24, 128.88, 126.94, 126.39, 125.98, 118.84, 105.53 (10 × C-arom), 55.20 (OCH_3_), 45.80 (CH), 18.04 (CH_3_); DEPT: 10 × CH_3_, 10 × CH_2_, 12 × CH; MS (EI) *m/z =* 682.7 [M]^+^ (C_45_H_62_O_5_).

*Oleanoyl ketoprofenate* (**17**): after purification by silica gel column chromatography yield: 0.27 g (79%) of **17** (R_f_ = 0.32, hexane**/**EtOAc 4:1) as colorless resin (with a tendency to crystallize); IR ν_max_ (KBr, cm^−1^) = 3300–3420 (COOH), 1725 (C=O, ester ketopr-olean), 1690 (COOH), 1175 (C–O, ester ketopr-olean); ^1^H-NMR (CDCl_3_): Oleanoyl moiety: δ = 10.95 (1H, COOH), 5.27 (1H, t, *J* = 3.4, CH-12), 4.47 (1H, m, CH-3), 2.80 (1H, dd, *J* = 13.6, 3.6, CH-18), 1.21–0.61 (21H, s 7 × CH_3_); Ketoprofen moiety: δ = 7.80 (1H, d, *J* = 8.0, CH-arom), 7.73 (1H, s, CH-arom), 7.64 (1H, d, *J* = 7.2, CH-arom), 7.58 (1H, d, *J* = 6.8, CH-arom), 7.57 (1H, d, *J* = 6.0, CH-arom), 7.53 (1H, d, *J* = 6.4, CH-arom), 7.49 (1H, d, *J* = 7.6, CH-arom), 7.47 (1H, d, *J* = 8.8, CH-arom), 7.43 (1H, d, *J* = 7.6, CH-arom), 3.78 (2H, m, CH), 1.59 (3H, d, *J* = 7.2, CH_3_); ^13^C-NMR (CDCl_3_): Oleanoyl moiety: δ = 178.28 (C-28), 143.79 (C-13), 122.22 (C-12), 81.34 (C-3), 55.22 (C-5), 47.50 (C-9), 46.70 (C-17), 45.95 (C-19), 41.61 (C-18), 41.28 (C-14), 39.26 (C-8), 37.85 (C-1), 37.74 (C-4), 36.62 (C-10), 33.84 (C-21), 33.08 (C-29), 32.54 (C-7), 32.36 (C-22), 30.66 (C-20), 28.43 (C-15), 27.72 (C-23), 27.65 (C-2), 25.88 (C-27), 23.47 (C-30), 23.38 (C-16), 23.05 (C-11), 18.09 (C-6), 18.03 (C-26), 16.62 (C-24), 16.53 (C-25); Ketoprofen moiety: δ = 196.57 (C=O), 173.57 (C=O, ester ketopr-olean), 141.22, 140.95, 137.88, 137.57, 130.00, 129.26, 128.83, 128.44, 128.39, 128.28 (12 × C-arom), 45.83 (CH), 15.27 (CH_3_); DEPT: 8 × CH_3_, 10 × CH_2_, 15 × CH; MS (EI) *m/z =* 692.9 [M]^+^ (C_46_H_60_O_5_).

*Oleanoyl ketoprofenate methyl ester* (**18**): after purification by silica gel column chromatography yield: 0.31 g (88%) of **18** (R_f_ = 0.50, hexane**/**EtOAc 4:1) as colorless resin (with a tendency to crystallize); IR ν_max_ (KBr, cm^−1^) = 1725 (C=O, ester ketopr-olean), 1700 (COOCH_3_), 1185 (C–O, ester ketopr-olean); ^1^H-NMR (CDCl_3_): Oleanoyl moiety: δ = 5.27 (1H, t, *J* = 3.6, CH-12), 4.46 (1H, m, CH-3), 3.62 (3H, s, COOCH_3_), 2.81 (1H, dd, *J* = 13.6, 3.6, CH-18), 1.21–0.61 (21H, s 7 × CH_3_); Ketoprofen moiety: δ = 7.80 (1H, d, *J* = 8.0, CH-arom), 7.73 (1H, s, CH-arom), 7.64 (1H, d, *J* = 7.2, CH-arom), 7.58 (1H, d, *J* = 6.8, CH-arom), 7.57 (1H, d, *J* = 6.0, CH-arom), 7.53 (1H, d, *J* = 6.4, CH-arom), 7.49 (1H, d, *J* = 7.6, CH-arom), 7.47 (1H, d, *J* = 8.8, CH-arom), 7.43 (1H, d, *J* = 7.6, CH-arom), 3.78 (2H, m, CH), 1.59 (3H, d, *J* = 7.2, CH_3_); ^13^C-NMR (CDCl_3_): Oleanoyl moiety: δ = 178.28 (C-28), 143.79 (C-13), 122.22 (C-12), 81.34 (C-3), 55.22 (C-5), 51.49 (COOCH_3_), 47.50 (C-9), 46.70 (C-17), 45.95 (C-19), 41.61 (C-18), 41.28 (C-14), 39.26 (C-8), 37.85 (C-1), 37.74 (C-4), 36.62 (C-10), 33.84 (C-21), 33.08 (C-29), 32.54 (C-7), 32.36 (C-22), 30.66 (C-20), 28.43 (C-15), 27.72 (C-23), 27.65 (C-2), 25.88 (C-27), 23.47 (C-30), 23.38 (C-16), 23.05 (C-11), 18.09 (C-6), 18.03 (C-26), 16.62 (C-24), 16.53 (C-25); Ketoprofen moiety: δ = 196.57 (C=O), 173.57 (C=O, ester ketopr-olean), 141.22, 140.95, 137.88, 137.57, 130.00, 129.26, 128.83, 128.44, 128.39, 128.28 (12 × C-arom), 45.83 (CH), 15.27 (CH_3_); DEPT: 9 × CH_3_, 10 × CH_2_, 15 × CH; MS (EI) *m/z =* 706.9 [M]^+^ (C_47_H_62_O_5_).

#### 3.2.3. Synthesis of Iminoester-Type Hybrid Compounds **19**–**26**

Oleanolic acid oxime (**5**) or its methyl ester (**6**) (1 mmol), DCC (1.03 g, 5 mmol) and anti-inflammatory drugs: ibuprofen, aspirin, naproxen, or ketoprofen (4 mmol), were mixed at 0 °C and then stirred at room temperature in dry CHCl_3_ (12 mL) for 2 h. Hexane (8 mL) was added to the reaction mixture and cooled. Next, the resulting precipitate of dicyclohexylurea was filtered. The organic phase was washed with 5% aqueous hydrochloric acid, 5% aqueous sodium bicarbonate and water and dried over anhydrous sodium sulfate. After removal of the drying agent and solvent evaporation, the crude new iminoesters **19**–**26** were purified with column chromatography on silica gel using hexane–ethyl acetate (4:1, *v*/*v*) mixture as a eluent.

*Oleanoyl oxime ibuprofenate* (**19**): after purification by silica gel column chromatography yield: 0.46 g (74%) of **19** (R_f_ = 0.45; hexane**/**EtOAc 4:1) as colorless solid, mp: 163–165 °C; IR ν_max_ (KBr, cm^−1^) = 3300–3420 (COOH), 1715 (C=O, iminoester ibu-olean), 1695 (COOH), 1170 (C–O, iminoester ibu-olean); ^1^H-NMR (CDCl_3_): Oleanoyl moiety: δ = 11.17 (1H, COOH), 5.53 (1H, t, *J* = 3.4, CH-12), 2.84 (1H, dd, *J* = 13.4, 3.4, CH-18), 1.35–0.72 (21H, s, 7 × CH_3_); Ibuprofen moiety: δ = 7.18 (2H, d, *J* = 8.4, CH-arom), 7.08 (2H, d, *J* = 8.0, CH-arom), 3.85 (1H, m, CH), 2.43 (2H, d, *J* = 7.0, CH_2_), 2.25 (1H, m, CH), 1.55 (3H, d, *J* = 7.0, CH_3_), 0.89 (3H, s, CH_3_), 0.88 (3H, s CH_3_); ^13^C-NMR (CDCl_3_): δ = Oleanoyl moiety: δ = 178.30 (C-28), 172.60 (C-3), 143.53 (C-13), 126.60 (C-12), 55.30 (C-5), 49.50 (C-9), 47.61 (C-17), 45.89 (C-19), 41.74 (C-14), 41.30 (C-18), 39.58 (C-8), 38.10 (C-1), 37.92 (C-4), 36.92 (C-10), 33.15 (C-29), 32.54 (C-22), 33.86 (C-21), 32.72 (C-7), 30.73 (C-20), 29.82 (C-15), 27.95 (C-23), 27.80 (C-2), 25.95 (C-27), 23.65 (C-30), 23.58 (C-16), 23.43 (C-11), 18.20 (C-6), 17.80 (C-26), 16.86 (C-24), 15.30 (C-25); Ibuprofen moiety: δ = 174.76 (C=O iminoester ibu-olean), 140.52, 138.40, 137.48, 128.98, 126.88, 126.69 (6 × C-arom), 44.59 (CH_2_), 40.18 (CHCOO), 27.56 (CH(CH_3_)), 22.32 , 18.58, 15.09 (3 × CH_3_); DEPT: 10 × CH_3_, 11 × CH_2_, 10 × CH; MS (EI) *m/z =* 657.6 [M]^+^ (C_43_H_63_NO_4_).

*Oleanoyl oxime ibuprofenate methyl ester* (**20**): after purification by silica gel column chromatography yield: 0.51 g (77%) of **20** (R_f_ = 0.67, hexane**/**EtOAc 4:1) as colorless resin; IR ν_max_ (KBr, cm^−1^) = 1710 (C=O, iminoester ibu-olean), 1170 (C–O, iminoester ibu-olean); 1700 (COOCH_3_); ^1^H-NMR (CDCl_3_): Oleanoyl moiety: δ = 5.28 (1H, t, *J* = 3.4, CH-12), 3.62 (3H, s, OCH_3_), 2.84 (1H, dd, *J* = 13.7, 3.4, CH-18), 1.49–0.72 (21H, s, 7 × CH_3_); Ibuprofen moiety: δ = 7.23 (2H, d, *J* = 8.0, CH-arom), 7.08 (2H, d, *J* = 8.0, CH-arom), 3.85 (1H, m, CH), 2.45 (2H, d, *J* = 7.0, CH_2_), 2.25 (1H, m, CH), 1.55 (3H, d, *J* = 7.0, CH_3_), 0.88 (3H, s, CH_3_), 0.87 (3H, s, CH_3_); ^13^C-NMR (CDCl_3_): Oleanoyl moiety: δ = 178.30 (C-28), 172.44 (C-3), 143.90 (C-13), 122.30 (C-12), 55.30 (C-5), 51.59 (COOCH_3_), 49.51 (C-9), 47.65 (C-17), 45.90 (C-19), 41.72 (C-14), 40.19 (C-18), 39.58 (C-8), 38.19 (C-1), 37.90 (C-4), 36.69 (C-10), 33.10 (C-29), 32.51 (C-22), 33.81 (C-21), 32.78 (C-7), 30.27 (C-20), 29.68 (C-15), 27.99 (C-23), 27.58 (C-2), 25.69 (C-27), 23.86 (C-30), 23.59 (C-16), 23.45 (C-11), 18.29 (C-6), 17.38 (C-26), 16.88 (C-24), 15.39 (C-25); Ibuprofen moiety: δ = 175.78 (C=O iminoester ibu-olean), 140.56, 137.62, 137.48, 129.22, 127.35, 127.10, 44.50 (CH_2_), 40.19 (CHCOO), 27.52 (CH(CH_3_)), 22.30, 18.59, 15.20 (3 × CH_3_); DEPT: 11 × CH_3_, 11 × CH_2_, 10 × CH; MS (EI) *m/z =* 671.6 [M]^+^ (C_44_H_65_NO_4_).

*Oleanoyl oxime aspirinate* (**21**): after purification by silica gel column chromatography yield: 0.39 g (62%) of **21** (R_f_ = 0.39, hexane**/**EtOAc 4:1) as colorless solid, mp: 104–107 °C; IR ν_max_ (KBr, cm^−1^) = 3350 (COOH), 1750 (C=O iminoester asp-olean), 1710 (COOH), 1695 (C=O asp.), 1175 (C–O, iminoester asp-olean); ^1^H-NMR (CDCl_3_): Oleanoyl moiety: δ = 10.80 (1H, COOH), 5.32 (1H, t, *J* = 3.4, CH-12), 3.31 (1H, m, CH-18), 1.33–0.83 (21H, s, 7 × CH_3_); Aspirin moiety: δ = 7.50, 7.43, 7.22, 7.10 (4H, m, CH-arom), 2.35 (3H, s, CH_3_). ^13^C-NMR (CDCl_3_): Oleanoyl moiety: δ = 178.26 (C-28), 176.40 (C-3), 143.96 (C-13), 122.13 (C-12), 55.39 (C-5), 47.63 (C-9), 46.76 (C-17), 45.83 (C-19), 41.75 (C-14), 41.39 (C-18), 40.95 (C-8), 38.19 (C-1), 37.99 (C-4), 36.98 (C-10), 33.11 (C-29), 32.55 (C-22), 33.82 (C-21), 32.71 (C-7), 30.72 (C-20), 29.89 (C-15), 27.92 (C-23), 27.80 (C-2), 25.90 (C-27), 23.69 (C-30), 23.58 (C-16), 23.45 (C-11), 18.29 (C-6), 17.89 (C-26), 16.81 (C-24), 15.32 (C-25); Aspirin moiety: δ = 171.12 (C=O iminoester asp-olean), 165.54 (COOCH_3_), 154.04, 131.38, 129.58, 127.79, 125.92, 121.90 (6 × C-arom), 21.30 (CH_3_); DEPT: 8 × CH_3_, 10 × CH_2_, 8 × CH; MS (EI) *m/z =* 631. 9 [M]^+^ (C_39_H_53_NO_6_).

*Oleanoyl oxime aspirinate methyl ester* (**22**): after purification by silica gel columnchromatography yield: 0.42 g (67%) of **22** (R_f_ = 0.47, hexane**/**EtOAc 4:1) as colorless solid, mp: 179–180 °C; IR ν_max_ (KBr, cm^−1^) = 1750 (C=O asp), 1710 (COOCH_3)_, 1650 (C=O asp), 1175 (C-O iminoester asp-olean); ^1^H-NMR (CDCl_3_): Oleanoyl moiety: δ = 5.28 (1H, t, *J* = 3.4, CH-12), 3.63 (3H, s, COOCH_3_), 2.99 (1H, dd, *J* = 13.7, 3.4, CH-18), 1.20–0.77 (21H, s, 7 × CH_3_); Aspirin moiety: δ = 7.97, 7.57, 7.32, 7.14 (4H, m, CH-arom), 2.35 (3H, s, CH_3_); ^13^C-NMR (CDCl_3_): Oleanoyl moiety: δ = 178.26 (C-28), 176.40 (C-3), 143.96 (C-13), 122.13 (C-12), 55.78 (C-5), 51.59 (COOCH_3_), 47.14 (C-9), 46.72 (C-17), 45.79 (C-19), 41.73 (C-14), 41.61 (C-18), 41.32 (C-8), 39.29 (C-1), 38.74 (C-4), 36.99 (C-10), 33.82 (C-21), 33.09 (C-29), 32.32 (C-22), 31.67 (C-7), 30.68 (C-20), 30.36 (C-15), 27.64 (C-23), 27.12 (C-2), 26.07 (CH_3_), 25.85 (C-27), 24.32 (C-30), 23.61 (C-16), 23.45 (C-11), 19.88 (C-6), 18.96 (C-26), 16.80 (C-24), 15.11 (C-25); Aspirin moiety: δ = 171.12 (C=O iminoester asp-olean), 165.54 (COOCH_3_), 154.04, 131.39, 129.60, 127.80, 125.97, 121.99 (6 × Carom), 21.08 (CH_3_); DEPT: 9 × CH_3_, 10 × CH_2_, 8 × CH; MS (EI) *m/z =* 645. 9 [M]^+^ (C_40_H_55_NO_6_).

*Oleanoyl oxime naproxenate* *(***23**): after purification by silica gel column chromatography yield: 0.55 g (81%) of **23** (R_f_ = 0.36, hexane**/**EtOAc 4:1) as colorless solid, mp: 160–162 °C; IR ν_max_ (KBr, cm^−1^) = 3300–3420 (COOH), 1725 (C=O, iminoester napx-olean), 1690 (COOH), 1175 (C–O, iminoester napx-olean); ^1^H-NMR (CDCl_3_): Oleanoyl moiety: δ = 9.78 (1H, COOH), 5.59 (1H, t, *J* = 3.4, CH-12), 2.50 (1H, dd, *J* = 13.6, 4.0, CH-18), 1.32–0.57 (21H, s, 7 × CH_3_); Naproxen moiety: δ = 7.70 (1H, d, *J* = 8.8, CH-arom), 7.68 (1H, s, CH-arom), 7.64 (1H, d, *J* = 8.8, CH-arom), 7.38 (2H, dd, *J* = 8.4, 8.4 CH-arom), 7.15 (1H, d, *J* = 11, CH-arom), 3.91 (3H, s, OCH_3_), 3.85 (1H, m, CH), 1.52 (3H, d, *J* = 7.0, CH_3_); ^13^C-NMR (CDCl_3_): Oleanoyl moiety: δ = 178.25 (C-28), 175.60 (C-3), 143.5 (C-13), 125.65 (C-12), 55.68 (C-5), 49.92 (C-9), 46.51 (C-17), 45.83 (C-19), 41.55 (C-14), 41.05 (C-18), 39.26 (C-8), 38.02 (C-1), 37.87 (C-4), 36.94 (C-10), 33.80 (C-21), 33.14 (C-29), 32.66 (C-7), 32.38 (C-22), 30.44 (C-20), 29.10 (C-15), 26.28 (C-23), 26.12 (C-2), 25.38 (C-27), 24.60 (C-30), 23.89 (C-16), 23.39 (C-11), 18.03 (C-6), 17.10 (C-26), 16.56 (C-24), 15.32 (C-25); Naproxen moiety: δ = 174.18 (C=O iminoester napx-olean), 154.06, 136.69, 133.51, 129.12, 128.92, 127.46, 125.99, 125.65, 119.07, 105.52 (10 × C-arom), 55.28 (OCH_3_); 45.52 (CH), 18.12 (CH_3_); DEPT: 9 × CH_3_, 10 × CH_2_, 11 × CH; MS (EI) *m/z =* 681.9 [M]^+^ (C_44_H_59_NO_5_).

*Oleanoyl oxime naproxenate methyl ester* (**24**): after purification by silica gel column chromatography yield: 0.52 g (75%) of **24** (R_f_ = 0.51, hexane**/**EtOAc 4:1) as colorless solid, mp: 110–112 °C; IR ν_max_ (KBr, cm^−1^) = 1710 (C=O, iminoester ibu-olean), 1695 (COOCH_3_), 1170 (C–O, iminoester ibu-olean); ^1^H-NMR (CDCl_3_): Oleanoyl moiety: δ = 5.27 (1H, t, *J* = 3.6, CH-12), 3.62 (3H, s, OCH_3_), 2.86 (1H, dd, *J* = 13.6, 4.2, CH-18), 1.23–0.73 (21H, s, 7 × CH_3_); Naproxen moiety: δ = 7.70 (1H, d, *J* = 8.8, CH-arom), 7.69 (1H, s, CH-arom), 7.42 (1H, d, *J* = 8.4, CH-arom), 7.39 (1H, d, *J* = 8.4, CH-arom), 7.14 (1H, d, *J* = 11, CH-arom), 3.91 (3H, s, OCH_3_), 4.04 (1H, m, CH), 1.59 (3H, d, *J* = 7.0, CH_3_); ^13^C-NMR (CDCl_3_): Oleanoyl moiety: δ = 178.26 (C-28), 172.51 (C-3), 143.93 (C-13), 122.23 (C-12), 55.77 (C-5), 51.59 (COOCH_3_), 49.91 (C-17), 46.69 (C-9), 45.79 (C-19), 41.45 (C-14), 41.30 (C-18), 39.24 (C-8), 38.60 (C-1), 37.90 (C-4), 36.9 (C-10), 33.80 (C-29), 33.08 (C-22), 32.90 (C-21), 32.42 (C-7), 32.31 (C-23), 31.16 (C-20), 30.68 (C-15), 27.60 (C-2), 26.93 (C-27), 25.31 (C-30), 23.60 (C-16), 22.99 (C-11), 18.87 (C-6), 18.76 (C-26), 16.76 (C-24), 15.01 (C-25); Naproxen moiety: δ = 175.73 (C=O iminoester napx-olean), 157.59, 136.74, 133.64, 129.16, 128.96, 127.49, 126.38, 125.99, 118.85, 105.58 (10 × C-arom), 55.30 (OCH_3_), 45.59 (CH), 18.75 (CH_3_); DEPT: 10 × CH_3_, 10 × CH_2_, 11 × CH; MS (EI) *m/z =* 695.9 [M]^+^ (C_45_H_61_NO_5_).

*Oleanoyl oxime ketoprofenate* (**25**): after purification by silica gel column chromatography yield: 0.26 g (73%) of **25** (R_f_ = 0.18; hexane**/**EtOAc 4:1) as colorless resin; IR ν_max_ (KBr, cm^−1^) = 3300–3420 (COOH), 1715 (C=O, iminoester ketopr-olean), 1695 (COOH), 1170 (C–O, iminoester ketopr-olean); ^1^H-NMR (CDCl_3_): Oleanoyl moiety: δ = 11.05 (1H, COOH), 5.28 (1H, s, CH-12), 2.84 (1H, dd, *J* = 13.4, 3.4, CH-18), 1.25–0.74 (21H, s, 7 × CH_3_); Ketoprofen moiety: δ = 7.80 (1H, d, *J* = 8.0, CH-arom.), 7.75 (1H, s, CH-arom.), 7.64 (1H, d, *J* = 7.2, CH-arom.), 7.58 (1H, d, *J* = 6.8, CH-arom.), 7.57 (1H, d, *J* = 6.0, CH-arom.), 7.53 (1H, d, *J* = 6.4, CH-arom.), 7.49 (1H, d, *J* = 7.6, CH-arom), 7.47 (1H, d, *J* = 8.8, CH-arom.), 7.43 (1H, d, *J* = 7.6, CH-arom.), 3.75 (2H, m, CH), 1.60 (3H, d, *J* = 7.2, CH_3_); ^13^C-NMR (CDCl_3_): Oleanoyl moiety: δ = 178.25 (C-28), 171.94 (C-3), 143.90 (C-13), 122.00 (C-12), 55.77 (C-5), 47.07 (C-9) 46.72 (C-17), 44.46 (C-19), 41.70 (C-18), 41.32 (C-14), 39.27 (C-8), 38.66 (C-1), 38.61 (C-4), 36.93 (C-10), 33.83 (C-21), 33.08 (C-29), 32.61 (C-7), 32.32 (C-22), 30.68 (C-20), 27.62 (C-23), 26.96 (C-15), 25.83 (C-2), 24.67 (C-27), 23.62 (C-30), 23.42 (C-16), 23.02 (C-11), 19.44 (C-6), 18.41 (C-26), 16.78 (C-24), 15.15 (C-25); Ketoprofen moiety: δ = 196.46 (C=O), 175.93 (C=O, ester ketopr-olean), 140.66, 140.59, 137.89, 137.48, 132.49, 131.59, 130.04, 129.35, 129.21, 128.99, 128.52, 128.31 (12 × C-arom), 45.52 (CH), 15.05 (CH_3_); DEPT: 8 × CH_3_, 10 × CH_2_, 14 × CH; MS (EI) *m/z =* 705.9 [M]^+^ (C_46_H_59_NO_5_).

*Oleanoyl oxime ketoprofenate methyl ester* (**26**): after purification by silica gel column chromatography yield: 0.30 g (84%) of **26** (R_f_ = 0.38, hexane**/**EtOAc 4:1) as colorless resin; IR ν_max_ (KBr, cm^−1^) = 1710 (C=O, iminoester ketopr-olean), 1170 (C–O, iminoester ketopr-olean); 1700 (COOCH_3_); ^1^H-NMR (CDCl_3_): Oleanoyl moiety: δ = 5.28 (1H, s, CH-12), 3.62 (3H, s, OCH_3_), 2.84 (1H, dd, *J* = 13.4, 3.3, CH-18), 1.27–0.74 (21H, s, 7 × CH_3_); Ketoprofen moiety: δ = 7.80 (1H, d, *J* = 8.0, CH-arom.), 7.75 (1H, s, CH-arom.), 7.64 (1H, d, *J* = 7.2, CH-arom.), 7.58 (1H, d, *J* = 6.8, CH-arom.), 7.57 (1H, d, *J* = 6.0, CH-arom.), 7.53 (1H, d, *J* = 6.4, CH-arom.), 7.49 (1H, d, *J* = 7.6, CH-arom), 7.47 (1H, d, *J* = 8.8, CH-arom.), 7.43 (1H, d, *J* = 7.6, CH-arom.), 3.75 (2H, m, CH), 1.60 (3H, d, *J* = 7.2, CH_3_); ^13^C-NMR (CDCl_3_): Oleanoyl moiety: δ = 178.25 (C-28), 171.84 (C-3), 143.92 (C-13), 122.02 (C-12), 55.77 (C-5), 51.52 (COOCH_3_), 47.07 (C-9) 46.72 (C-17), 44.46 (C-19), 41.70 (C-18), 41.32 (C-14), 39.27 (C-8), 38.66 (C-1), 38.61 (C-4), 36.93 (C-10), 33.83 (C-21), 33.08 (C-29), 32.61 (C-7), 32.32 (C-22), 30.68 (C-20), 27.62 (C-23), 26.96 (C-15), 25.83 (C-2), 24.67 (C-27), 23.62 (C-30), 23.42 (C-16), 23.02 (C-11), 19.44 (C-6), 18.41 (C-26), 16.78 (C-24), 15.05 (C-25); Ketoprofen moiety: δ = 196.46 (C=O), 175.93 (C=O, ester ketopr-olean), 140.66, 140.59, 137.89, 137.48, 132.49, 131.59, 130.04, 129.35, 129.21, 128.99, 128.52, 128.31 (12 × C-arom), 45.82 (CH), 15.02 (CH_3_); DEPT: 9 × CH_3_, 10 × CH_2_, 14 × CH; MS (EI) *m/z =* 719.9 [M]^+^ (C_47_H_61_NO_5_).

### 3.3. Evaluation of Hydrolytic Stability

The mixture of hybrid compound (esters: **11**, **13**, **15**, **17** and iminoesters: **19**, **21**, **23**, **25**) (0.1 mmol) and ethanol solution of HCl (pH = 5, 10 mL) was stirred at room temperature. After 1, 5, and 24 h, the mixture was sampled and analyzed using the TLC method with the use of hexane**/**EtOAc (4:1, *v*/*v*) as a eluent. The mixture was stirred at room temperature and next sampled and analyzed using the TLC method after 1, 5, and 24 h, respectively. Since all of the compounds were stable in the test at room temperature, another test was conducted at an elevated temperature (50 °C), and then sampled and analyzed using the TLC method after 1, 5, and 24 h respectively.

## 4. Conclusions

Starting from naturally-occurring oleanolic acid, its oxime, and their methyl esters, as well as from four synthetic anti-inflammatory drugs (ibuprofen, aspirin, naproxen, ketoprofen), 16 new derivatives were synthesized, combining the oleanolic acid active skeleton and anti-inflammatory structure into new single-hybrid chemical individuals. Oleanolic acid and its methyl ester were reacted with NSAIDs containing a non-blocked COOH group in the presence of DCC and DMAP in towards ester-type hybrids, while oleanolic oxime and its methyl ester were reacted with NSAIDs in the presence of DCC only in towards to iminoester-type hybrids. The structure of compounds obtained was confirmed by the spectral method. The presence of some functional groups in the terpene skeleton and NSAID structures could improve their potential biological activity. The resulting derivatives were determined and analyzed by the PASS method and they have great potential to become effective pharmaceutical individuals as anti-inflammatory, chemopreventive, hypolipemic, antisecretoric, antinociceptive, antiulcerative, or antineoplastic agents, various stimulants, inhibitor, antagonists, promoters, or regulators. Ester and iminoester moieties in these molecules are also a useful strategy for designing new therapeutic compounds and it should be noted that ester-type derivatives are more active agents using the PASS method. Ester connections exhibit very high probability of the occurrence of biological activity greater than 70%. A preliminary stability test showed that the resulting structures (**11**–**26**) are stable and not susceptible to degradation under acidic hydrolysis conditions. Furthermore, the importance and potential application of synthetic oleanolic acid derivatives are highlighted and the prospects for research on oleanolic acid are given. The hybridization method of natural and synthetic compounds is one of the most promising and fundamentally novel approaches for the design of new lead structures and the discovery of new and potent drugs in the field of medicinal chemistry.

## Figures and Tables

**Table 1:** Predicted activity of ester-type derivatives **11**–**18** determined by the PASS method.

Activity/Compounds Number	P_a_ Factor of Compounds 11–18 (P_a_ > 0.7, P_i_ ≤ 0.005)							
	11	12	13	14	15	16	17	18
Insulin promoter	0.983	0.968	0.966	0.926	0.948	0.900	0.965	0.921
Hepatoprotectant	0.926	0.925	0.957	0.956	0.929	0.924	0.891	0.889
Lipid metabolism regulator	0.927	0.874	0.969	0.958	0.952	0.930	0.966	0.953
Chemopreventive	0.925	0.918	0.967	0.965	0.938	0.923	0.864	0.855
Oxidoreductase inhibitor	0.921	0.893	0.907	0.878	0.903	0.878	0.924	0.895
Membrane integrity antagonist	0.912	0.889	0.926	0.908	0.904	0.878	0.892	0.859
Transcription factor NF kappa B stimulant	0.905	0.874	0.911	0.882	0.884	0.858	0.902	0.870
Transcription factor stimulant	0.905	0.874	0.911	0.882	0.884	0.858	0.902	0.870
Mucomembranous protector	0.903	0.811	0.877	0.783	0.868	0.799	0.875	0.782
Anti-inflammatory	0.877	0.872	0.841	0.835	0.860	0.861	0.890	0.885
Non-steroidal-anti-inflammatory agent	0.740	0.735	0.715	0.710	0.738	0.719	0.794	0.789
Hypolipemic	0.870	0.828	0.866	0.824	0.842	0.792	0.869	0.827
Antisecretoric	0.850	0.868	0.766	0.788	0.816	0.847	0.798	0.823
Antinociceptive	0.836	0.795	0.862	0.819	0.867	0.822	0.822	0.780
Antiulcerative	0.833	0.782	0.858	0.818	0.799	0.742	0.814	0.758
Antineoplastic	0.758	0.765	0.848	0.770	0.789	0.798	0.803	0.808
Caspase 3 stimulant	0.815	0.799	0.977	0.974	0.962	0.934	0.792	0.775
Caspase 8 stimulant	0.761	0.759	0.861	0.859	0.859	0.849	0.771	0.768
Apoptosis agonist	0.809	0.806	0.863	0.861	0.810	0.811	0.818	0.815

**Table 2:** Predicted activity of iminoester-type derivatives **19**–**26** determined by the PASS method.

Activity/Compounds Number	P_a_ Factor of Compounds 19–26 (P_a_ > 0.5, P_i_ ≤ 0.005)							
	19	20	21	22	23	24	25	26
Insulin promoter	0.942	0.853	0.846	-	0.760	-	0.842	-
Hepatoprotectant	0.702	0.706	0.743	0.741	0.717	0.701	-	-
Chemopreventive	-	-	0.727	0.717	-	-	-	-
Oxidoreductase inhibitor	0.836	0.734	0.831	0.721	0.776	-	0.841	0.744
Membrane integrity antagonist	0.815	0.718	0.867	0.813	0.789	-	0.719	-
Transcription factor NF kappa B stimulant	0.881	0.835	0.884	0.843	0.851	0.815	0.877	0.829
Transcription factor stimulant	0.881	0.835	0.884	0.843	0.851	0.815	0.877	0.829
Anti-inflammatory	0.776	0.762	0.725	0.711	0.751	0.748	0.802	0.790
Antinociceptive	0.700	-	-	-	0.742	-	-	-
Caspase 3 stimulant	-	-	0.830	0.815	0.806	0.757	-	-
Caspase 8 stimulant	-	-	0.752	0.749	0.716	-	-	-
Apoptosis agonist	-	-	0.790	0.786	-	-	-	-

“-”—potential activity in the P_a_ factor range of 50%–70%.
